# Acceptability and effectiveness of empathy-based provider training and community-level awareness activities on self-injectable contraceptive use in Niger, Lagos, and Oyo States, Nigeria: a mixed methods program evaluation

**DOI:** 10.1186/s12905-025-03992-w

**Published:** 2025-09-09

**Authors:** Susan Ontiri, Claire W. Rothschild, Fauzia Tariq, Oluwaseun Adeleke, Michael Titus, Mopelola Raji, Julius Njogu, Susannah Gibbs, Robin Swearingen, Fidelis Edet

**Affiliations:** 1 Population Services International, Washington, D.C. USA; 2 Population Services International, Nairobi, Kenya; 3 Society for Family Health-Nigeria, Abuja, Nigeria; 4 Present Address: International Center for Reproductive Health, Nairobi, Kenya

**Keywords:** DMPA-SC, Depot medroxyprogesterone acetate-subcutaneous, Self-care, Self-injection, Injectable contraception, Empathy

## Abstract

**Background:**

Interventions aimed to increase healthcare provider empathy and capacity to deliver person-centered care have been shown to improve healthcare seeking and outcomes. In the context of self-injectable contraception, empathetic counseling and coaching may be promising approaches for addressing “fear of the needle” among clients interested in using subcutaneous depot medroxyprogesterone (DMPA-SC). In Nigeria, the Delivering Innovation in Self-Care (DISC) project developed and evaluated an empathy-based in-service training and supportive supervision intervention for public sector family (FP) planning providers implemented in conjunction with community-based mobilization.

**Methods:**

The DISC intervention was evaluated using a quasi-experimental mixed methods design. Effectiveness of the intervention on DMPA-SC service delivery, including self-injection (SI) and provider administration, was assessed using a single-group interrupted time series design that leveraged phased implementation in 36 facilities. Service delivery data were extracted from the Nigerian Health Information System for the pre-intervention period and using program monitoring tools for the intervention and post-training maintenance period. Outcomes were modeled using linear generalized estimating equations. In-depth interviews were conducted with trained providers to assess acceptability and perceived changes in SI attitudes and behaviors.

**Results:**

Mean DMPA-SC service provision increased by 28.1 visits on average per facility in the first month of implementation, relative to a pre-intervention data strengthening phase (95% confidence interval [CI] 18.0–38.3). The intervention was associated with an increase in mean facility-level SI service delivery of 25.9 visits (95% CI 16.3–35.4). The intervention was associated with overall increases in FP service delivery. Increases in DMPA-SC service provision were sustained in the post-training maintenance period. In qualitative interviews, trained providers reported increased client demand for SI, coupled with increased provider confidence to counsel and train clients to self-inject. While providers indicated that stockouts of intramuscular DMPA (DMPA-IM) resulted in shifts towards DMPA-SC, we did not observe decreases in DMPA-IM or long-acting reversible contraception provision in the quantitative data.

**Discussion:**

Our findings demonstrate the effectiveness and acceptability of a program combining supply- and demand-side interventions aimed at expanding awareness and access to self-injectable contraception in Nigeria. In this context, providers highly valued in-service training and ongoing support that built capacity for empathetic client engagement.

**Supplementary Information:**

The online version contains supplementary material available at 10.1186/s12905-025-03992-w.

## Background

Discrimination, stigma, and lack of empathetic care are well-documented barriers to sexual and reproductive health (SRH) care-seeking and utilization [1]. There is ample evidence that healthcare providers can act as gatekeepers of contraceptive services [2], limiting access by refusing to provide services or by creating a health facility environment that is stigmatizing to groups such as adolescents and unmarried women [1, 3]. Subcutaneous depot medroxyprogesterone acetate (DMPA-SC) is a method of contraception designed to be self-injectable (SI). The pharmaceutical formulation is the same as its intramuscular alternative (DMPA-IM); however, dosage of SC is slightly lower per unit compared to IM. Within the group of DMPA injectables, women have three options: 1) DMPA-SC self-injection (SI), 2) provider-administered (PA) DMPA-SC; or 3) DMPA-IM, which is not designed for self-administration. As a contraceptive self-care method [4], DMPA-SC SI has the potential to reduce barriers to contraceptive use, particularly in settings with limited health workforce and for populations for whom repeated facility-based care-seeking is otherwise challenging or undesirable, including those facing stigma or who wish to use a contraceptive method covertly [5]. One feature that makes DMPA-SC SI unique among its DMPA alternatives is the flexibility for users to inject themselves outside of the health facility setting.

Lack of provider competency and training, in addition to provider-imposed barriers, may limit women’s ability to access DMPA-SC for SI. Studies in various settings have found that provider-imposed restrictions on DMPA-SC SI use are common, particularly for specific groups of women such as adolescents, youth, and women with lower educational attainment [2, 6]. Although feasibility of self-injection has been demonstrated among young women and those with low levels of education [7], providers in many settings continue to express concerns about the safety of self-injection in these groups [8]. Other healthcare providers report a lack of confidence providing training for self-injection as a reason for restricting access to DMPA-SC [9, 10]. Evidence suggests that interventions focused on person-centered care may improve provider–client interactions and clients’ experiences of contraceptive counseling and use. For example, in Malawi, providers trained to provide DMPA-SC for SI have noted that empathetic care alongside step-by-step demonstration can greatly alleviate clients’ fear of needles and improve clients’ confidence in self-injecting [11]. Taken together, these findings suggest that interventions to reduce provider biases and to facilitate interpersonal connection during the SI training process may be an effective strategy for increasing access to and acceptability of DMPA-SC SI.

In Nigeria, injectables and implants are the most utilized modern contraceptive methods, each comprising one-fourth of total modern contraceptive method use nationally [12]. DMPA-SC was introduced through private health channels in 2015, with public sector rollout beginning in 2016 [13]. Nigeria moved quickly to develop national DMPA-SC guidelines and policies, including a five-year “roadmap” to scale DMPA-SC nationally [13]. Despite the supportive policy environment, awareness of DMPA-SC SI remains low: While nationally representative information on knowledge, awareness, and practice of DMPA-SC SI are not yet available, the Performance Monitoring for Action (PMA) program estimates that – despite high awareness of injectable contraception – only 21% of women of reproductive age in both Kano and Lagos States had ever heard of DMPA-SC SI as of 2020–2021 [14]. As a result, DMPA-SC comprises just 9.6% and less than 1% of the contraceptive method mix in Kano and Lagos, respectively [15].

The Delivering Innovation in Self-Care (DISC) project, implemented by Population Services International, Society for Family Health-Nigeria, and other consortium partners, supports the delivery of SRH self-care technologies and practices in Uganda, Nigeria, and Malawi. DISC’s model combines community sensitization and mobilization activities with healthcare provider capacitation, with the goal of increasing awareness of and service readiness for SRH self-care, with a focus on DMPA-SC SI. This model is based on evidence that programs that simultaneously address both supply- and demand-side factors – such as increasing community awareness and strengthening community-facility linkages – have proven effective for increasing SRH service utilization [16], quality of care [17], and equitable access [18, 19]. We conducted a mixed-method quasi-experimental evaluation to assess the effectiveness of the training in combination with community-based awareness and mobilization activities on DMPA-SC SI uptake and to assess the acceptability and self-reported changes in attitudes and practices towards SI after the training from the perspective of participating healthcare providers.

## Methods

### Study design and population

This mixed-methods program evaluation was conducted in Lagos, Oyo, and Niger States of Nigeria from January 2021 through October 2023. These states were selected due to their status as high priority geographies for the DISC program, with high levels of program implementation intensity. Lagos and Oyo States, located in the southwest region, are characterized by the large urban and peri-urban populations of the Lagos and Ibadan metropolitan areas, respectively, while Niger State in the north central region is primarily rural. Modern contraceptive prevalence varies substantially across these regions, ranging from 6.4% in Niger to 22.2% in Oyo and 29.4% in Lagos among married women of reproductive age [12]. Contraceptive injectables are the most prevalent modern method used in Oyo and Niger States, while male condoms are the predominant modern method in Lagos State [12]. Nationally, injectable provision is concentrated in the public sector, with three-fourths of injectable users reporting their most recent method source was a public hospital or health center [12].

The quantitative component of the study consisted of an assessment of group-level changes in DMPA-SC SI service provision in 36 health facilities (12 per state). Qualitative in-depth interviews with providers were conducted to better understand providers’ experiences of the training and subsequent provision of contraceptive care and counseling, as well as to contextualize quantitative findings. Within each state, study facilities were selected from within local government areas (LGAs) with no major recent security incidents and where no DISC activities had been conducted, to ensure a valid pre-implementation baseline (Phase 0). Within eligible LGAs, study facilities were selected that offered the full range of contraceptive methods, had the highest reported contraceptive injectable service volumes in the prior 12 months, and had low but non-zero baseline DMPA-SC service provision and low SI provision, as reported in the Nigerian Health Management Information System (NHMIS). A total of 110 providers were trained across the 36 selected study facilities, with an average of 3 providers trained per facility.

### The DISC intervention

**Fig. 1:**
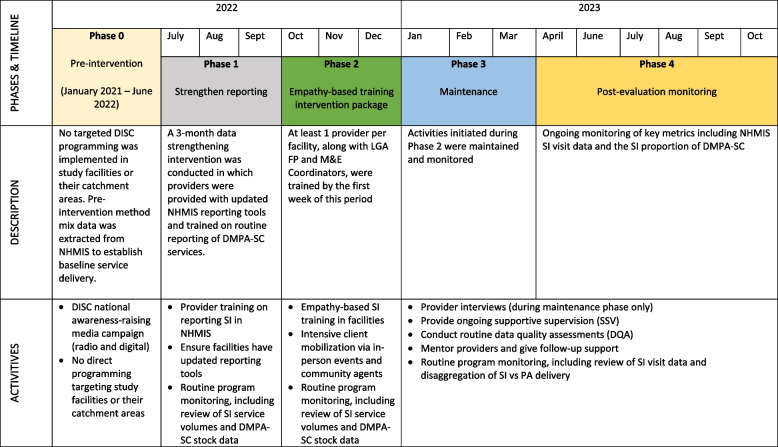
Description of Phased Implementation of the DISC Project

The DISC intervention model was designed based on learnings from formative research, insight generation, and evidence-based prototyping with prospective SI clients and family planning (FP) providers that the project conducted throughout 2020–2021 [20]. The intervention package includes a provider-focused empathy-based training; community mobilization and awareness-raising; and post-training support (Fig. 1).

#### Provider empathy-based training

The training directly addressed client drop-off during initiation of self-injection by strengthening provider skills to provide empathetic counseling, with the goal of increasing client confidence and addressing common concerns such as fear of the needle and pain. Training modules – which included tools such as checklists and job aids – address aspects of quality service provision including values clarification, informed choice, person-centered care, proficiency in self-injection, approaches to strengthening supportive supervision, and strategies to improve commodity forecasting and reporting. DISC’s approach to empathy training builds upon behavior change principles (Supplemental Material, Figure S1), with content tailored in a way that addresses both providers’ motivations and capabilities and builds in practice components and supportive supervision and on-job mentorship to reinforce learnings (Supplemental Material, Figure S2). Trainings were conducted at study facilities and were co-facilitated by representatives from the Federal Ministry of Health, State Primary Healthcare Development Agency (state FP and M&E officers), and DISC-Nigeria project staff. Integrated within the training is a practicum which allowed trainees to visit a clinic where clients have been pre-mobilized to seek DMPA-SC services, so that each trainee was able to practice DMPA-SC SI counseling in a supervised setting. During the practicum, each trainee provided actual SI training to a client who has expressed interest in the method and provided consent to take part in a training exercise. The provider practicum was observed by one of the trainers, who used a checklist to note down any feedback during the training session. The trainer is instructed to only interrupt counseling if incorrect, incomplete, or harmful information is being passed along to the client—to ensure each client receives quality counseling. Trainers provided feedback to all trainees afterwards in a plenary session.

#### Community mobilization and awareness-raising

Commencing shortly before the training delivery and continuing concurrently, the DISC-Nigeria team led facility- and community-based events aiming to counter misinformation, educate community members about self-care and FP with the aid of a structured flipchart, and raise awareness about the available SI services at nearby health facilities participating in the study. Community mobilizers also aim to bring care to the community-level by organizing community outreach events with the presence of trained service providers at bustling locations where women frequently gather or work – such as health centers on clinic days, markets, women groups/trade associations, village squares, and religious centers. Awareness-raising activities, including digital interventions such as interactive voice response and a digital companion (chatbot), made use of DISC’s #DiscoverYourPower (DYP) slogan and leveraged DISC’s campaign messages which had been co-designed with a creative agency. Concurrently, radio campaigns were underway in the three states, as were social media ad campaigns on Facebook. Additional detail about the DYP campaign and the program’s awareness-raising activities have been published elsewhere [21, 22].

#### Post-training support

The DISC empathy-based SI materials included a supportive supervision checklist to help supervisors assess resource availability and providers’ adherence to empathetic counseling practices. Post-training supportive supervision was provided jointly by reproductive health coordinators (RHCs), project staff and state social and behavior change officers to ensure high quality service delivery, support for empathetic counseling skills and supply management, and accuracy of data capture. Following trainings, health facilities received ongoing on-site mentoring support to reinforce knowledge and skills learned during trainings. The onsite mentorship also helped cascade the training to 22 other providers who were not part of the original training. Each post-training visit included an evaluation of the competency of SI services, a review of community mobilization activities, data quality checks, and commodity availability tracking. Additionally, ongoing community mobilization activities were conducted in facilities’ catchment areas during follow-up visits.

### Quantitative methods

To evaluate the impact of the intervention on DMPA-SC service provision within study facilities, we used a single-group interrupted time series analysis (ITSA) design that leveraged phased implementation. This design allows for detection of both immediate changes (measured as change in mean service provision at the facility-level in the first month of each implementation phase compared to the last month in the prior phase), as well as long-term changes (measured as changes in the monthly trend in average facility-level service provision relative to the monthly trend in the preceding phase).

The following four phases were defined for the purpose of the interrupted time series analysis:

- Phase 0: Pre-intervention (January 2021 – June 2022). This phase captured baseline service delivery in the absence of DISC intervention. No DISC project activities were implemented in study facilities or the surrounding LGAs during this phase. Monthly contraceptive service statistics data were extracted from the NHMIS.
- Phase 1: Data strengthening (July – September 2022). The aim of the data strengthening phase was two-fold: first, to ensure that pre-implementation data reported in study facilities were high quality and provide a valid pre-implementation estimate of service delivery; and second, to allow the evaluation approach to differentiate the impact of the intervention itself from improvements in data quality. To these aims, DISC held one-day-long SI data reporting trainings at the state level during the first week of July 2022, which were attended by at least one FP provider from each of the 36 facilities, the facility data reporting officer, and LGA monitoring and evaluation (M&E) officers who are responsible for data reporting into that national NHMIS. The objective of this training was to equip providers on how to correctly document data on DMPA-SC/SI for reporting into NHMIS. Trained providers subsequently cascaded the training to other providers in the study facilities.
- Phase 2: Implementation (October – December 2022). DISC project implementation consisted of a combined supply- and demand-side approach, details of which are provided in the previous section.
- Phase 3: Maintenance and Routine Monitoring (January 2022 – October 2023). The maintenance phase (through March 2023) included ongoing mentorship and supportive supervision, while during the routine monitoring phase (April through October 2023) mentorship ended and only routine monitoring activities continued.

The ITSA approach was selected for its suitability for capturing outcomes with the ability to change rapidly without a long lag time [23]. For each phase of the implementation tested here, activities were conducted in full (for the data strengthening training and the provider empathy-based training) or initiated and ongoing (for the community-based awareness and mobilization activities) in the first week of the respective phase. It was expected that training, combined with community mobilization, could be reasonably expected to impact service delivery volumes in the short-term. For this reason, assessing level change in the first month of implementation was deemed appropriate to capture short-term changes associated with program implementation activities. In addition to measuring short-term “level” changes in the first month of the phase, the ITSA also allows for assessment of longer-term and/or lagged program effects through the estimation of differences in month-on-month trends in the current relative to the previous phase.

We hypothesized that implementation of the empathy-based training intervention would result in increases in the level of DMPA-SC SI service provision, comparing Phase 1 to Phase 2. In the maintenance phase, we hypothesized that both levels and trends observed in Phase 3 would be maintained relative to Phase 2.

### Data sources

NHMIS data were extracted at the facility level for each month of interest during Phase 0. Per NHMIS reporting protocol, facilities that did not deliver a specific method in a month left NHMIS data on that method’s service delivery volumes missing. As a result, all missing facility-month observations in Phase 0 for specific FP method service volumes were assumed to be zero (i.e., no method provision). However, it is possible that some instances of missingness may be due to non-reporting or data completeness issues. To limit bias due to non-reporting, we excluded all NHMIS data available prior to January 2021 due to suspected incompleteness and quality issues with DMPA-SC reporting prior to this date.

In Phases 1–3, data were extracted by DISC data entry clerks every month directly from facility family planning registers and submitted to project monitoring and evaluation (M&E) staff monthly via Excel reporting template for review and analysis. For these data, zeros were used to report no service delivery for a specific facility/month. To assess data completeness, we tabulated instances of missing observations and observed few (*n* = 8) instances of missing data in Phases 1–3, all for facilities that reported non-missing data on service provision for at least 1 contraceptive method for that month. For that reason, we also treat these instances of missingness as zeros.

### Primary and secondary outcomes of interest

Study facilities recorded the number of FP visits in which clients received DMPA-SC by mode of administration: for SI visits, the client self-injected under provider observation, while for PA visits the injectable was administered by the provider. Our three primary outcomes of interest were number of DMPA-SC SI visits; number of DMPA-SC PA visits; and number of total DMPA-SC visits (SI and PA combined).

To evaluate whether implementation was associated with shifts in overall FP service volumes and in the service delivery method mix, we conducted secondary analyses using analogous ITSA models on total FP, long-acting reversible contraception (LARC), and DMPA-IM visits. To model total FP visits, we summed FP visits for all methods except condoms, as data on condom distribution in unreliable and inconsistently reported across facilities. LARC visits were calculated by summing all intrauterine device (IUD) and implant service provision. All outcomes were modeled as service delivery totals aggregated at the facility-month level.

### Statistical analysis

We exploited the phased implementation to evaluate trends in DMPA-SC service provision at study facilities using a single-group ITSA. Models were fit using generalized estimating equation (GEE) models accounting for repeated observations at the facility level. Facility-level clustering allows unbiased estimation of standard errors with anticipated non-independence of observations within the same facility. We used linear models with observations at the facility-month-level, in which coefficients can be interpreted as mean differences in monthly facility-level counts (e.g., of DMPA-SC SI visits). Models were fit with an exchangeable working correlation structure and robust standard errors. Linear models were selected for ease of interpretation and for their emphasis on potential public health impact. Robust standard errors allow for unbiased statistical inference even in the presence of anticipated model misspecification due to non-Normal distribution of the count outcomes. We analyzed models that were both unadjusted and adjusted for season (in 3-month-long periods) and LGA using dummy variables. Accounting for seasonality is recommended in time series analysis; in the context of healthcare utilization, healthcare utilization is often driven by seasonal patterns, which can bias interrupted time series analyses with short intervals when seasons differs by intervention period [23]. Dummy variables for LGA account for unmeasured, time-invariant heterogeneity between groups. All estimates were generated using the *xtitsa* package in the statistical analysis software Stata [24].

### Sensitivity and exploratory analyses

Because we were not able to conduct a complete audit of facility registers and client intake forms during Phase 0, pre-intervention data was extracted directly from NHMIS. The project team opted to use data collectors to extract monthly data directly from facility registers during Phase 1–3 because of concerns around the quality of service provision data once it was aggregated into the NHMIS monthly summary forms. To assess robustness of our results to this shift to use of programmatic data, we conducted a sensitivity analysis in which we refit models using NHMIS data only. We also conducted a sensitivity analysis in which Phases 0 and 1 were combined into a single pre-intervention phase, which allows for a larger number of observations in the interval immediately prior to the intervention used to estimate level and trend changes associated with Phase 2. In an exploratory analysis, we estimated primary outcomes in state-specific models.

### Qualitative methods

In January 2023, we conducted in-depth interviews with 31 RHCs and front-line family planning service providers purposively selected in Niger, Lagos, and Oyo. Interviews were conducted to assess the acceptability and self-reported changes in attitudes and behaviors related to SI services among providers who had received the empathy training intervention.

Prior to selection of providers for the interviews, SI visit service data for the period between April and December 2022 were reviewed for all 36 DISC-supported evaluation facilities. Three performance categories were defined: facilities with increased, decreased, or stable SI service provision after the intervention. A subset of 18 health facilities were purposively selected across the performance categories to sample 24 providers (9 in Niger and Lagos each, and 6 in Oyo). A sample of 24 providers and 7 RHCs was expected to be sufficient to achieve the required theoretical saturation for generation of insights relevant for contextualizing the quantitative evaluation findings [25].

Trained research assistants conducted interviews in English using a semi-structured guide, which was developed for this study (Supplemental Material, Interview Guides) Interviews were audio-recorded with verbal consent of the participating providers and transcribed verbatim under the supervision of the first and third authors [SO, OA].

All interview transcripts were reviewed for quality and uploaded into Dedoose, a cloud-based application for managing and analyzing qualitative data [26]. A hybrid approach of inductive and deductive coding was used to analyze the data [27]. All the transcripts were read in full by one co-author (JN) to gain deeper understanding of the interview scope and content and uncover emerging subcodes for documentation in the final coding structure. Each interview was reviewed line-by-line, and relevant segments and phrases labelled with respective codes and subcodes. Dedoose was then used to assess data patterns and identify recurrent themes. De-identified quotations from the participants are reported in this paper to characterize analyzed themes.

#### Ethics statement

NHMIS data were collected at the facility-month level and did not include any individual patient identifiers. After consultation with the Nigerian Federal Ministry of Health, data in this manuscript are reported at the state level to protect the confidentiality of individual health facilities.

Permission was sought from the facility in-charge prior to provider eligibility screening. Healthcare providers provided verbal consent for eligibility screening. Eligible providers were read an informed consent form by the study enumerator. Informed consent for participation was documented via paper-based enumerator signature and date on an informed consent acknowledgment form. Providers did not receive any incentive or compensation for participation in the study.

All study procedures were approved by the Population Services International (PSI) Research Ethics Board and Nigerian National Health Research Ethics Committee (NHREC/01/01/2007–24/05/2021).

## Results

### Pre-intervention and data strengthening phases

At the beginning of Phase 0 (pre-intervention), study facilities reported an average of 11.6 DMPA-SC visits per month (95% confidence interval [CI] 8.0, 15.3), of which 1.5 visits (95% CI 0.6–2.5) were SI (Table 1A).

**Table 1:** Interrupted time series analyses on primary outcomes

Panel A. Unadjusted Models			
	(Model 1)	(Model 2)	(Model 3)
	DMPA-SC SI	DMPA-SC PA	DMPA-SC (SI and PA)
Phase 0 trend	0.09	0.29	0.38*
	(−0.01—0.18)	(−0.01—0.60)	(0.08—0.68)
Level change, Phase 1 vs. 0	2.02*	−4.70*	−2.69
	(0.17—3.87)	(−8.57—−0.84)	(−7.06—1.69)
Trend change, Phase 1 vs. 0	−1.28	−0.28	−1.56
	(−2.64—0.08)	(−2.60—2.04)	(−4.15—1.03)
Level change, phase 2 vs. 1	25.92**	3.71	29.63**
	(16.89—34.96)	(−1.48—8.91)	(20.42—38.85)
Trend change, phase 2 vs. 1	2.60	−3.68*	−1.08
	(−0.88—6.08)	(−6.82—−0.54)	(−5.64—3.47)
Level change, phase 3 vs. 2	4.22	1.17	5.39
	(−2.82—11.25)	(−4.00—6.34)	(−3.08—13.85)
Trend change, Phase 3 vs. 2	−0.28	4.15**	3.87
	(−3.69—3.13)	(1.84—6.46)	(−0.15—7.90)
Phase 0 level	1.54**	10.10**	11.64**
	(0.57—2.51)	(6.71—13.49)	(7.95—15.33)
Observations	1,224	1,224	1,224
Number of facilities	36	36	36
**Panel B. Adjusted Models**			
	(Model 1)	(Model 2)	(Model 3)
	DMPA-SC SI	DMPA-SC PA	DMPA-SC (SI and PA)
Phase 0 trend	0.14**	0.30	0.45**
	(0.04—0.25)	(−0.01—0.61)	(0.14—0.75)
Level change, Phase 1 vs. 0	1.18	−3.73	−2.55
	(−1.20—3.56)	(−8.12—0.66)	(−7.66—2.56)
Trend change, Phase 1 vs. 0	−1.34	−0.29	−1.63
	(−2.71—0.03)	(−2.61—2.03)	(−4.21—0.96)
Level change, phase 2 vs. 1	25.85**	2.29	28.14**
	(16.33—35.37)	(−3.49—8.07)	(17.97—38.30)
Trend change, phase 2 vs. 1	2.60	−3.68*	−1.08
	(−0.88—6.08)	(−6.82—−0.54)	(−5.64—3.47)
Level change, phase 3 vs. 2	4.04	1.02	5.07
	(−2.86—10.95)	(−4.54—6.58)	(−3.27—13.40)
Trend change, Phase 3 vs. 2	−0.17	4.31**	4.14*
	(−3.57—3.22)	(1.95—6.67)	(0.06—8.22)
Phase 0 level	−0.41	14.79**	14.38**
	(−4.91—4.09)	(10.44—19.13)	(6.54—22.21)
Observations	1,224	1,224	1,224
Number of facilities	36	36	36

All models are linear GEE models with robust standard errors adjusted for clustering at the facility level and using an exchangeable working correlation structure. Adjusted models include variables for season (modeled as dummy variables indicating January-March, April-June, July–September, and October-December) and local governmental authority (LGA). Robust 95% confidence interval in parentheses. Trends are interpreted as the mean month-on-month change in the outcome at the facility-level. “Phase 0 level” estimates refer to the model’s y-intercept, which can be interpreted as the predicted value of the outcome in the first month of the Phase 0 (pre-intervention) period

^**^ *p* < 0.01, * *p* < 0.05

This low level of DMPA-SC self-injection was consistent with findings from qualitative interviews, in which participants explained that most DMPA-SC provision prior to the empathy training was provider-administered, with uptake of SI services impeded by multiple barriers. Many providers cited clients’ “fear of the needle” and fear of associated risks including abscesses as predominant barriers to SI uptake.

*My reaction was that most of our clients will not accept it, because some of them… you ask them to take ordinary TT [tetanus toxoid vaccination] they will run away... So, we were thinking then that some of them will not accept it because of the fear of injection. Primary Health Center (PHC) Provider #2*

Some of the providers were concerned that allowing clients to self-inject would lead to “abuse and quackery”, given that women would be playing the role of trained health workers.

*When I first heard about self-inject, I was afraid that patient may abuse that knowledge like the way do things, like when I have headache and take paracetamol... So, I was afraid of such abuse in the sense that someone after mastering it, may go home and become a health worker in their own home. PHC Provider #2*

There were also mixed reports regarding the availability of DMPA-SC over a period of 9 months preceding data collection; some facilities reported steady supply while others had experienced stock-outs. Providers reported several strategies that they used to mitigate DMPA-SC stock-outs, including commodity forecasting and early ordering, borrowing from other facilities, and receiving new supplies from FP Coordinators.

**Fig. 2:**
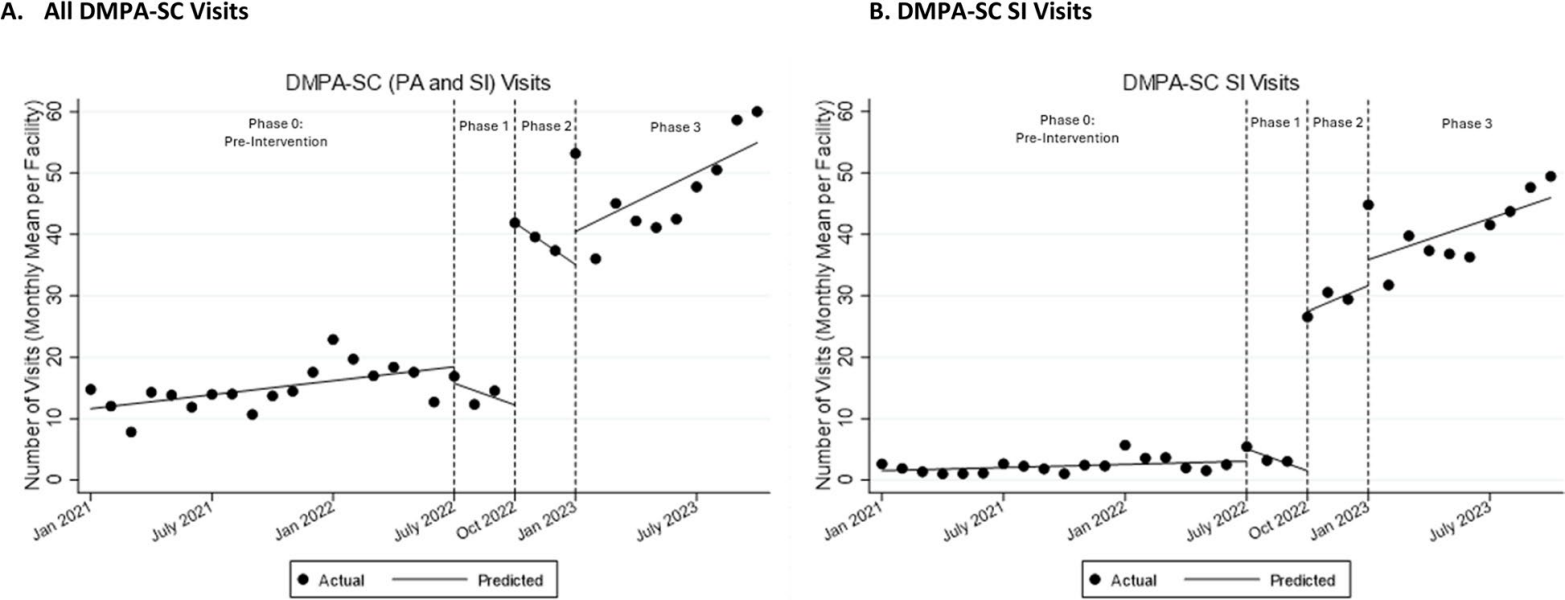
Interrupted times series analysis of total DMPA-SC and DMPA-SC SI service provision, by implementation phase. Notes: Figures present observed data points and predicted values from unadjusted models

Comparing Phase 0 to Phase 1, we observe a small increase in reported provision of DMPA-SC SI in the unadjusted model by 2.0 (95% CI 0.2–3.9) visits on average per facility in the first month of Phase 1, but no difference in the adjusted model (Table 1B, Fig. 2). We observe a decrease in DMPA-SC PA delivery by an average of 4.7 visits (95% CI −8.6—-0.8) per facility in the first month of Phase 1, but no difference in the adjusted model. There were no differences observed in the first month of Phase 1 in total number of DMPA-SC visits. No other changes in monthly trend comparing Phase 1 to Phase 0 were detected.

### Provider assessment of the empathy-based training

Overall, providers indicated that the empathy-based training content was useful, enjoyable, and interactive. The practicum was the most appreciated aspect of the training.

*I like everything about the training. In fact, we had practical sessions where we did video recording, and everybody was cracking jokes with patients... By taking us to the center, we gained more experience. So, I like it, there’s nothing I didn't like there. It was interesting. PHC Provider #14*

Didactic training was also found to be relevant and useful, particularly the focus on strengthening providers’ counseling skills to address side effect concerns related to SI.

*During the training they elaborate more on the rumor. For us that we should let them know that all what they are saying is rumor, and they should please take it and see and when they do, that what “Taye” is saying about family planning might not happen to “Kehinde”, so they should try and take their own if any side effect happened to them, they should not go to other place, they should come back to the provider that attended to them... PHC Provider #15*

Providers highlighted that the empathy training addressed implicit biases from the provider perspective, which may have previously restricted provision of SI services to only specific categories of women – such as those whom providers perceived to be well educated or residing in urban settings.

*…What was apparent among the health workers was the kind of skepticism especially where the client is not a literate person, so there was that holding back. But for the literate ones, there was no restriction or holding back. So, one of the learning at that training was that education was not a limiting factor. Once you are able to properly demonstrate the procedure to client, and teach them on how to properly dispose, they can actually do it on their own. FP Coordinator #2*

No participants reported disliking any aspects of the empathy training. However, participants had several suggestions for improving or modifying the training. Several providers felt that expanding the training to encompass provision of empathetic, person-centered care for the full range of contraceptive methods would be valuable. Others suggested that post-training follow-up strategies could be strengthened, such as refresher trainings and reflection meetings between trainers and trained providers to review provider capacity and develop strategies to strengthen capacity gaps.

### Self-injection services after the empathy-based training

In the quantitative analysis, we found increases in the first month of program implementation across DMPA-SC SI and all DMPA-SC provision in both unadjusted and adjusted models. There was no difference in the first month of the program implementation in DMPA-SC PA delivery. Focusing on adjusted estimates, we found that mean DMPA-SC service provision increased by 28.1 (95% CI 18.0–38.3) visits per facility in the first month of Phase 2. The observed increase was driven by SI visits, which increased by 25.9 (95% CI 16.3–35.4) visits per facility. We did not detect significant differences in the monthly trend in DMPA-SC or SI service provision relative to Phase 1.

Most providers felt that the training improved their confidence in their ability to counsel and train clients to SI. Providers specifically cited that the training had supported them to adopt effective empathy-based strategies fostering positive client-provider relationships.

*You know other FP methods you just give it to them when the patient comes but teaching your client [SI] has taken the relationship to another level. The rapport now is more cordial and a bit informal because of the teaching that comes in between. I may give a patient OCP [Oral Contraceptive Pill] or implant and I may not even remember the patient if we meet on the road. But because I teach, it makes every contact special. PHC Provider #2*

*The counseling aspect gets to the heart of the client. After that, the environment, the way they treat the client, step by step how they make them comfortable, they are relaxed to be able to speak to their inner minds. These were the things we are taught in the training you know. It has really helped me to improve, and to be able to give time to my clients. You know some of them when they come are not even decisive on what to do. By the time you come asking them especially those that are not learned, their mind is off especially on the aspect of all these taboos... PHC Provider #13*

Many providers also described an increase in client demand for SI services after the DISC empathy training and community mobilization intervention. Shifts in client demand were associated with increased community mobilization efforts bolstering client awareness of SI family planning services, positive community norms around DMPA-SC, and higher provider capacity and willingness to address client’s related needs and concerns around SI. Providers noted there had been increased awareness raising and mobilization activities around DISC supported health facilities’ catchment areas, leading to increases in the uptake of SI services.

*Yes [before empathy training], we were trained but that time women were not rushing, but now women they are rushing because formerly they don’t have the knowledge maybe due to lack of sensitization. PHC Provider #12*

Providers indicated that higher demand of DMPA-SC was being used to update product forecasts and product planning to ensure future availability. When DMPA-SC was stocked out at the facilities, providers resorted to taking several actions including offering clients a different available method at the facility, asking clients to come back again, or directing them to purchase the product or method elsewhere. Alternatively, FP Coordinators cited stockout of DMPA-IM as a reason for increased demand for DMPA-SC, with former IM clients shifting to SC. However, providers indicated that clients were satisfied with their experiences when they switched to SI from other method types. In addition, many providers felt that clients – specifically experienced injectable users – preferred SI for its convenience and perceptions that it was less painful.

*Some people who are into the older methods do switch over to SI especially because of time saving... Most people on injectables are switching to SI more than those on implant... But we have not seen someone on SI switching over to any other method. PHC Provider #3*

Some providers indicated that some DMPA-SC users were coming to the facility for provider-administered injection for reasons including covert use and forgetting how to SI by the time of reinjection.

*Secondly, some will say my husband does not know that I came for this [DMPA-SC]. So how am I expected to take the injection home and how will my husband love me? PHC Provider #16*

In both adjusted and unadjusted quantitative models, we estimate a significant decrease in the monthly trend of DMPA-SC PA service provision from no monthly change in Phase 1 (adjusted β [aβ]: 0.01; 95% CI: −2.23–2.25) to a monthly decrease of 3.7 visits on average per facility per month in Phase 2 (aβ: −3.7, 95% CI −6.0-−1.4). Several providers described efforts to discourage ongoing DMPA-SC usage as a provider-administered method.

*There are some clients that have been on Sayana Press [DMPA-SC]. They like it, they don’t want to change, and they don’t want to administer it themselves. So, they come back to the facility anytime they are due. Like I said the idea is that it is a “do it yourself” procedure, and it is something that we will encourage our clients to do on their own. FP Coordinator #2*

### Maintenance phase

There was no evidence of changes in any of the primary outcomes in the first month of the maintenance phase relative to Phase 2. However, there was some evidence of changes in monthly trend comparing Phase 3 to Phase 2: in adjusted models, we found a small increased trend in DMPA-SC (PA and SI) visits (by 4.1 visits per facility per month [95% CI 0.06–8.2]) but no difference in trend in unadjusted models. In both adjusted and unadjusted models, we estimate an increased trend in DMPA-SC PA in Phase 3 vs. Phase 2. Focusing on adjusted results, we estimate a month-on-month increasing trend in DMPA-SC PA service provision by 0.6 visits on average per facility per month in Phase 3 (95% CI: 0.2, 1.1), relative to a monthly decrease in average DMPA-SC PA provision by 3.7 visits per facility per month (95% CI: −6.0- −1.4) in Phase 2.

These modest longer term quantitative findings could be in part explained by providers’ perceptions of impacts of providing self-injectable DMPA-SC on workloads and client waiting times***.*** Several providers reported that they felt that providing counseling on SI was unrealistic for providers with heavy workloads, citing the additional time taken for counseling and SI training relative to providing the injection themselves:

*For other provider maybe when they are busy, they feel they don’t want to waste more time in counseling, their busy schedule and the staff shortage, they just want to do it and move on to another thing to avoid wasting time. FP Manager #1*

This perception that SI training is prohibitively time consuming, particularly in contexts with high service volumes, may explain the increasing trend towards DMPA-SC PA as intervention intensity reduced. Qualitative findings were mixed, however, with other providers perceiving SI as having the potential to reduce burdens on health workers and health facilities as a form of task-sharing with clients, and to have time-savings benefits for women who opted to SI.

### Secondary outcomes and sensitivity analyses

**Fig. 3:**
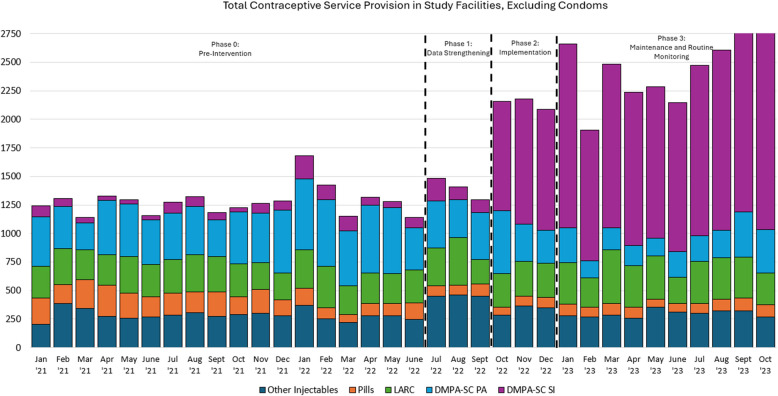
Family Planning Service Provision in Study Facilities, by Month and Method Type. Notes: Total visits are reported as the sum of all FP service delivery provided by study facilities (*n* = 36), by month and method type

We observed a monthly average of 1,279 and 1,395 total FP visits conducted across all 36 study facilities in Phase 0 (pre-intervention) and Phase 1 (data strengthening) phases, respectively (Fig. 3). FP service delivery volumes increased to an average of 2,140 monthly visits in Phase 2 (intervention) and 2,451 monthly visits in Phase 3 (maintenance). The average monthly share of all FP service provision that was DMPA-SC SI increased from 7% in the pre-intervention phase to 46% in the intervention phase (data not shown).

We found no evidence of differences in level or monthly trend of all FP visits comparing Phase 1 to Phase 0 (Table 2 and Supplement, Figure S3). We found an increase in overall method provision of 27.5 FP visits per facility in the first month of Phase 2 (implementation) (95% CI 12.2–42.7), but no change in monthly trend compared to Phase 1 (data strengthening). In Phase 3 (maintenance) we found no evidence of change in level or trend in all FP visits compared to Phase 2. We found no differences in level or trend across the evaluation phases in LARC provision. For DMPA-IM provision, we estimate an increase of 3.6 (95% CI 0.4, 6.7) DMPA-IM visits in the first month of Phase 1 (data strengthening); we did not find evidence of any other changes in level or trend in DMPA-IM service provision across implementation phases.

**Table 2:** ITSA estimates on secondary outcomes

	(Model 1)	(Model 2)	(Model 3)
	All Methods Excluding Condoms	LARC	DMPA-IM
Phase 0 trend	0.18	0.01	−0.09
	(−0.25—0.61)	(−0.10—0.12)	(−0.22—0.04)
Level change, Phase 1 vs. 0	3.54	1.65	3.56*
	(−3.16—10.24)	(−0.94—4.24)	(0.39—6.74)
Trend change, Phase 1 vs. 0	−2.76	−1.67	0.41
	(−7.29—1.76)	(−3.72—0.39)	(−1.50—2.32)
Level change, phase 2 vs. 1	27.49**	4.40	−2.92
	(12.23—42.74)	(−0.87—9.67)	(−7.66—1.83)
Trend change, phase 2 vs. 1	1.60	1.81	0.22
	(−5.07—8.26)	(−0.59—4.21)	(−2.02—2.46)
Level change, phase 3 vs. 2	2.35	0.07	−1.61
	(−6.70—11.40)	(−3.71—3.85)	(−4.35—1.14)
Trend change, Phase 3 vs. 2	2.79	−0.25	−0.60
	(−2.17—7.75)	(−1.56—1.07)	(−1.61—0.42)
Phase 0 level	32.20**	8.37*	1.96*
	(15.71—48.69)	(0.61—16.13)	(0.40—3.52)
Observations	1,224	1,224	1,224
Number of facilities	36	36	36

All models are linear GEE models with robust standard errors adjusted for clustering at the facility level and using an exchangeable working correlation structure. Models include adjustment variables for season (modeled as dummy variables indicating January-March, April-June, July–September, and October-December) and local governmental authority (LGA). Robust 95% confidence interval in parentheses. Trends are interpreted as the mean month-on-month change in the outcome at the facility-level. “Phase 0 level” estimates refer to the model’s y-intercept, which can be interpreted as the predicted value of the outcome in the first month of the Phase 0 (pre-intervention) period

^**^ *p* < 0.01, * *p* < 0.05

Findings were robust to reanalysis using the NHMIS-only data (Supplemental Material, Table S1 and Figure S4). Results were also robust to an alternative specification combining Phases 0 and 1 into a single pre-intervention phase (Supplemental Material, Table S2). In state-level ITSA, we observed statistically significant positive increases within each individual State in overall DMPA-SC and in DPMA-SC SI visits in the first month of the implementation phase (Supplemental Material, Table S3).

## Discussion

Evidence across a variety of health areas and settings has demonstrated that interventions focused on improving the relationship between health workers and clients can effectively increase provider empathy and confidence and improve healthcare utilization and other outcomes [28–31]. Yet such approaches have not, to our knowledge, been widely used in settings such as Nigeria to support provider capacity to deliver a newly introduced self-injectable contraceptive method. The DISC project, which combined community-based mobilization with empathy-based training and supervision for providers, was associated with a substantial increase in the level of DMPA-SC self-injection. Total DMPA-SC service provision increased substantially, from an average of 535 visits per month in the pre-intervention phase to over 1,400 visits per month in the intervention phase across the 36 study facilities – three-fourths of which were self-injected. Estimates comparing the full-intensity intervention phase with a lighter-touch maintenance and extended routine monitoring phase suggest that program effects were sustained in the nearly year-long period following program roll-out.

DISC’s demand- and supply-side intervention components were implemented concurrently. Increases in overall FP service delivery associated with DISC implementation, without concurrent decreases in LARC or DMPA-IM provision, suggest that results were driven by increased DMPA-SC service provision rather than by shifts in the method mix at pre-intervention service volumes. This finding implies that community-based sensitization and outreach activities were effective at increasing service seeking for DMPA-SC. Shifts in the relative delivery of DMPA-SC from predominantly PA in the pre-intervention period to SI in the intervention and maintenance phases suggest that DISC’s provider-focused training and supervision also effectively expanded SI behaviors among DMPA-SC users. These findings are consistent with our qualitative findings, which highlight low client demand and low provider capacity to support SI in the pre-intervention period.

It is likely that increased demand – in conjunction with increased provider capacity to address any latent demand for SI that was present prior to community-based mobilization efforts – contributed to the observed increases in SI delivery. However, concurrent delivery of the supply- and demand-side intervention components prevents disentanglement of the relative contributions of each. In the context of very low baseline awareness and uptake of a newly introduced method such as DMPA-SC in Nigeria [14], such community-based activities are essential for ensuring that service providers receiving didactic instruction also have access to DMPA-SC clients to complete the practical component of the training. It was this combination of instruction with observed practice that was highlighted by many interviewed providers as most valuable for solidifying learnings and confidence to provide SI, and corresponds with best practice in clinical instruction (see, for example, [32]). While the effectiveness of the empathy-based provider training would be difficult to assess in a context with very low demand for SI, it remains of interest to what extent the empathy training intervention alone would have shifted service delivery.

Our qualitative findings highlight high acceptability of the DISC empathy training intervention among participating healthcare providers. Provided in a context characterized by very little baseline self-injection of DMPA-SC within study facilities, the training was viewed by healthcare providers as successful in improving interpersonal counseling and care for self-injection and reducing provider biases that served as barriers to access. Providers also noted pre-training biases that had previously limited their provision of DMPA-SC – and SI specifically – to more highly educated and urban women. While most providers had received previous “hard skills” training or sensitization on provision of DMPA-SC, including via SI, providers noted that the DISC training provided concrete examples and practice on interpersonal care and counseling that were critical for them to effectively coach women to SI; several providers even reported amazement in their ability after the DISC training to effectively establish rapport and to empathetically address clients’ questions and concerns. Another key success of the empathy training was in convincing providers that SI should not be restricted by sociodemographic characteristics of the client, thereby improving free contraceptive choice. Future research is required to evaluate whether similar empathy-based approaches improve equitability of service delivery, an outcome which was beyond the scope of this evaluation. Qualitative findings are consistent with our quantitative results, which demonstrate that most pre-intervention DMPA-SC provision was provider-administered. While the DISC training focused on self-injectable contraception, qualitative findings emphasize health providers’ interest in expanding “soft skills” components of FP training curricula across the full range of FP methods and counseling areas.

While Nigerian policy and guidelines do not restrict DMPA-SC use to SI, a few trained providers noted that they believed DMPA-SC should be used for SI only. Future research is needed to better understand drivers of provider-administered DMPA-SC. Providers in our study described provider-administered DMPA-SC use as occurring in the context of overburdened providers or in the case of stockouts of alternative contraceptive injectable methods; the latter practice is consistent with Nigerian policy, which advises provision of DMPA methods as “interchangeable” if one formulation (-IM or -SC) is out of stock [33]. It is plausible that some clients interested in SI may receive provider-administered DMPA-SC prior to deciding to self-inject at a later reinjection date. It is poorly understood whether and why some clients may prefer to use DMPA-SC PA on an ongoing basis, relative to the less-costly DMPA-IM. Such research will be valuable for refining DMPA-SC policies and guidance, which should ideally seek to maximize client choice while balancing considerations of cost to the health system.

Rapid increases in service delivery for a specific method, or substantial shifts in the service provision method mix, raise concerns about the potential for provider bias, particularly in the context of programs emphasizing service delivery of a specific or limited range of methods. DISC’s training was designed to enable providers to effectively offer DMPA-SC to clients interested in contraceptive injectables, and to support SI among women choosing DMPA-SC. The program conducted routine monitoring of service delivery method mix as a strategy for flagging potential concerns. Routine monitoring of indicators of contraceptive autonomy and coercion are critical for all FP programs, but particularly for those that support introduction and scaled delivery of a single or limited range of contraceptive methods. Further work to establish best practices for programmatic monitoring and adaptive management to ensure full, free, and informed contraceptive choice [34] is needed.

Several systems-related challenges beyond the scope of the DISC project were highlighted as potential quality gaps and provide important context to these findings. Commodity and supply issues beyond the scope of the DISC project were cited as drivers of reduced method availability across the range of available injectable contraceptive methods. Several providers cited stockouts of DMPA-IM leading to higher DMPA-SC uptake, although we did not observe evidence of changes in levels or trends in average facility-level DMPA-IM service provision. Systems strengthening activities, including appropriate forecasting and supply of all commodities, are critical for reducing true commodity shortages [35].

This study has several strengths. The quasi-experimental ITSA design allows for robust inference of program effectiveness, particularly in the context of a well-defined intervention such as this. The phased implementation of data strengthening activities prior to the core DISC intervention allows us to differentiate impacts of the intervention from improvements in reporting and data quality alone.

The study also has several limitations. The short time periods within the data strengthening and program implementation phases of just 3 months may reduce the reliability of regression coefficient estimates and does not allow for assessment of longer-term effects within each phase of implementation. While some recommend 3 as the minimum number of time points per phase in an ITSA, others recommend at least 6 for reliability of estimated regression coefficients [36]. In addition, the short time periods raise concern that potential lagged effects of phased interventions may be captured (and therefore attributed to) later phases of implementation. In our data, the magnitude of the observed shifts in delivery volumes corresponding to the defined period of the full-intensity intervention roll out alleviate some of these concerns. Facilities were selected purposively based on several factors, including having relatively high DMPA-SC PA but low DMPA-SC SI service volumes in the pre-intervention period and location in an area with relatively high security. Results are therefore not expected to be widely generalizable. Finally, this study qualitative data from providers with facility-based service statistics data. DMPA-SC SI reported in the context of this study occurred in the facility under provider-observation. It is beyond the scope of this study to evaluate the impacts of the intervention on longer-term contraceptive behaviors, such as receipt of additional DMPA-SC reinjection units and subsequent SI outside of the facility.

This study provides novel insights into the effectiveness and acceptability of an empathy-focused intervention implemented in Nigeria to expand access and quality provision of DMPA-SC self-injection. In the context of DISC’s programming in Nigeria, an intervention grounded in principles of empathy and person-centered quality of care provides a promising blueprint for expanding access to contraceptive self-care.

## Supplementary Information

Supplementary Material 1.

Supplementary Material 2.

## Data Availability

De-identified provider interview transcripts are available upon reasonable request to the authors. De-identified service statistics data are available upon reasonable request to the authors and with permission of the Federal Ministry of Health of Nigeria.

## Abbreviations

CI: Confidence interval
DISC: Delivering Innovation in Self-Care
DMPA-IM: Intramuscular depot medroxyprogesterone
DMPA-SC: Subcutaneous depot medroxyprogesterone
DYP: “Discover Your Power”
FP: Family planning
GEE: Generalized estimating equation
ITSA: Interrupted time series analysis
LARC: Long-acting reversible contraception
LGA: Local government area
M&E: Monitoring and evaluation
NHMIS: Nigerian Health Management Information System
PA: Provider administration; provider-administered
PHC: Primary health center
PSI: Population Services International
RHC: Reproductive Health Coordinator
SI: Self-injection
SRH: Sexual and reproductive health
