# Supplementary material for: Acceptability and effectiveness of empathy-based provider training and community-level awareness activities on self-injectable contraceptive use in Niger, Lagos, and Oyo States, Nigeria: a mixed methods program evaluation
Source: BMC Womens Health. 2025 Sep 9;25(Suppl 1):428. doi: 10.1186/s12905-025-03992-w (PMC12421749; doi:10.1186/s12905-025-03992-w)
Supplement: Supplementary file 1 — Supplementary Material 1. [file 12905_2025_3992_MOESM1_ESM.docx]

***DISC Moment of Truth Study Evaluation***

***Stakeholders-RHC Coordinators – Interview Guide***

| ***Purpose:*** *The purpose of this interview guide is to help facilitate discussion with RHC coordinators in the LGA and the respective State. The guide should be used flexibly with each RH Coordinator.*  *You must seek consent before you start the interview*  ***Form of data recording:*** *Notes of key points and quotes per topic area are handwritten by the facilitator into their data collection notebooks and then written out in more detail immediately following the data collection.* |
| --- |

***INTRODUCTION/*** *Preamble* (**to be read by the interviewer at the start of each encounter):**

*IAM ............ FROM.........*

*The purpose of this discussion/interview is to............*

*I would like to know if it is okay for us to proceed with the discussion. The expected time needed for this interview is about 1 hour*

Thank you for your time. As the facilitator, I will also be taking some notes to help me remember our discussion. Nothing captured will be followed on or held against you personally. Do you have any questions before we begin?

***Questions***

1. **When was DMPA SC rolled out in your facilities?**
2. **Have all facilities been trained on self-injectable contraceptives?**
   1. **If not, on average, what proportion of facilities have a trained provider who is able to counsel women and train them on self-inject**
3. **What is the attitude of providers in offering SI services to women?**
4. **How is the supply of DMPA SC contraceptive in your facilities?**
   1. Has the supply been consistent?
   2. How would you compare the supply with other contraceptive methods?
5. **Do you conduct supportive supervision of SI to providers?**
   1. What is the frequency?
   2. What content do you cover?
   3. What criteria do you use to select facilities to be visited?
6. **What are the current barriers to SI service provision?**
7. **What are your recommendations for providing SI services?**

State:

LGA:

Stakeholder Name:

Title:

**Thank you for your time.**

***DISC Moment of Truth Study Evaluation***

***Provider IDI 1 – Interview Guide***

| ***Purpose:*** *The purpose of this interview guide is to help facilitate discussion with health providers in the evaluation sites. The guide covers topic areas related to their experience of providing DMPA SC services to women, both self-inject and provider administered. The guide should be used flexibly with each health provider.*  *You must seek consent before you start the interview*  ***Form of data recording:*** *Notes of key points and quotes per topic area are handwritten by the facilitator into their data collection notebooks and then written out in more detail immediately following the data collection.* |
| --- |

***INTRODUCTION/*** *Preamble* (**to be read by the interviewer at the start of each encounter):**

*IAM ............ FROM.........*

*I would like to have some discussions with you in relation to your role as a service provider in this facility...........*

*The purpose of this discussion/interview is to............*

*I would like to know if it is okay for us to proceed with the discussion. The expected time needed for this interview is about 1 hour*

Thank you for your time. As the facilitator, I will also be taking some notes to help me remember our discussion. Nothing captured will be followed on or held against you personally. Do you have any questions before we begin?

***Topic area 1 – About the participant and their own experiences with providing contraceptives***

**I’d like to start by asking you a bit about your work as a health provider. I’ll also be asking about your own perceptions/beliefs and experiences with providing contraceptives to clients.**

**Please tell me about yourself?**

- How old are you?
- How long have you been in this position? Probe for qualification and experience.
- How long have you been counseling on and providing contraceptives to clients?
- How many providers offer family planning in this facility?
- How many providers were trained on MOT in this facility?
- Are they all still in this facility? - any transfers?
- Any cascade training on MoT done for other providers in the facility?

**If you think of instances in which you counselled clients on contraceptives, what informs your counselling (how you package information) and the types of methods you focus on?**

- How do you go about assessing your client’s contraceptive needs?
- How do you decide which contraceptive method might be best suited to your client?
- Which counseling approach do they use? (Balanced for counseling, C4C)

***Topic area 2 – Perceptions and experiences of self-injectable contraception***

**I’d now like to ask you what you think about self-injection as a method of contraception.**

**Tell me about your initial reaction when you heard about contraceptive self-injection for the first time.**

**Tell me about your perspective and experience on DMPA SC self-injecting contraceptives before DISC interventions**

1. When did you first learn about self-injecting DMPA SC contraceptives?
   1. Where did you hear about this method?
   2. What did you feel about self-inject contraception before MoT training?
   3. Did you use to allow women to self-inject during their first visit?
2. Had providers been trained on DMPA SC service provision?
   1. Did the training consist of self-inject component?
   2. If yes, what type of training had you received (classroom, on the job training)?
   3. Who trained you (organization/partner)?
3. Who records family planning data in this facility?
   1. Has that person been trained on recording SI?
4. Before DISC rolled-out demand generation activities, were there any activities being conducted in the facilities to encourage the uptake of SI services?
   1. If yes, what were some of those activities?
   2. Who was supporting those activities, partner, organization
5. What are some of the barriers to providing SI services in this facility before MoT training?

***Topic area 3 – Experiences of the self-inject training/mentorship***

**I’m now going to ask a few questions about the self-inject training/mentorship you recently received.**

**Thinking about your experience providing SI training to women:**

1. What kind of barriers have you encountered in trying to discuss self-inject contraception with clients before the MOT training?
   1. How did it change following the MOT training?
2. What have you found easy or difficult in introducing self-injection before MOT training?
3. What kind of support, if any, did you feel you needed to overcome some of the barriers you’ve experienced?
   1. Was the training effective in providing you the kind of support?
4. What concerns did women have on SI uptake before MOT training?
   - Was the training effective in helping you address the concerns that women had?

**Following the MOT training, do you feel that it was worthwhile to attend? Why/why not?**

1. What did you like most about the training - the topics discussed, the practicum, etc.?
2. Which aspects of the training did you find the most helpful/relevant to you?
3. Which aspects of the training were unnecessary/could have been better focused or used?
4. How can the training be improved to help you to be more effective in providing SI services.

**How has your experience of counseling women changed following the MOT training?**

1. How was this similar/different to how you’ve counseled clients in the past?
2. Is there a difference in how women react/respond?
3. What is the most common concern that clients express about using SI?
4. How confident are you to address these concerns?
5. What has changed regarding how women take up SI following the training? What about PA DMPA-SC?
6. In your experience, how does providing self-inject contraceptive services compare to other types of contraception available in the health service?

**Share experiences of the women whom you have counselled about self-injectable contraceptives or who have accessed the self-inject contraceptive method through you** (adolescents/teens, young adult women, older women; relationship status - married, dating, single). Is there a specific age group or group of women that prefer this method?

- - Why do you think this category prefer this method?

**Inquire into how the availability of commodity has affected DMPA-SC service provision:**

1. Describe DMPA-SC commodity availability over the last nine months?
2. If stock out is reported, how has that affected DMPA-SC service provision.
3. Do you provide women with additional doses to take home?
4. How does SI and PA service provision change based on commodity availability?
5. Share an experience about the episode of commodity stock out and how it was resolved?

**Partner support**

1. Before DISC, were there any partners that were supporting SI services in this facility?
   - If yes, who were the partners and what support did they provide (capacity building, mentorship, commodities, demand generation)
2. Who are the current partners supporting SI services at the moment?
   - What is the scope of their support (capacity building, mentorship, commodities, demand generation)

**Supportive Supervision**

1. Do you receive supportive supervision that is targeted at family planning?
2. What are about for SI services?
   1. If yes, who conducts the supportive supervision? (Partner/state)
   2. When was the last time it was done?

**In conclusion, do you feel any differently about self-injectable contraceptives after the training than you did before the training?** In what ways?

- What is the biggest change, if any, that you will be making or already have made in how you discuss/deliver contraceptives/self-inject, coming out of the training?
- What are things not addressed by the training that you still struggle with?

**Is there anything we haven't yet discussed that is important for me to know to understand your experience with contraceptive self-injectables?**

***Part B: Understanding SI performance following the training:***

**Category 2: Facilities that have not had a change:**

Looking at the trend of SI data we have observed that there has been little or no change following the MOT training.

- - In your opinion why do you think the SI visits have not changed following the training?
  - What are the contributing factors to the low demand of SI contraceptive methods? Do you consistently offer women SI? If not, why?
  - Has your facility been conducting demand-generation activities?
  - Are all the providers offering FP services trained on self-inject? Has there been instances of trained providers moving from the facility?
  - How has the data quality changed over time? Could there have been instances of overreporting of SI that was revised following the training? If yes, what changed?

Thank you for your time.
