# Supplementary material for: Acceptability and effectiveness of empathy-based provider training and community-level awareness activities on self-injectable contraceptive use in Niger, Lagos, and Oyo States, Nigeria: a mixed methods program evaluation
Source: BMC Womens Health. 2025 Sep 9;25(Suppl 1):428. doi: 10.1186/s12905-025-03992-w (PMC12421749; doi:10.1186/s12905-025-03992-w)
Supplement: Supplementary file 2 — Supplementary Material 2. [file 12905_2025_3992_MOESM2_ESM.docx]

**Acceptability and effectiveness of empathy-based provider training and community-level awareness activities on self-injectable contraceptive use in Niger, Lagos, and Oyo States, Nigeria: A mixed methods program evaluation**

**Supplemental Material**

**Table S1. Sensitivity analysis: ITSA estimates using NHMIS data only**

|  | (Model 1) | (Model 2) |
| --- | --- | --- |
|  | Mean Number of DMPA-SC Visits (PA and SI) | Mean Number of DMPA-SC SI Visits |
|  |  |  |
| Phase 0 trend | 0.25 | 0.10 |
|  | (-0.23 - 0.74) | (-0.21 - 0.41) |
| Level change, Phase 1 vs. 0 | -3.15 | 2.23 |
|  | (-9.62 - 3.32) | (-1.35 - 5.81) |
| Trend change, Phase 1 vs. 0 | -0.15 | -1.61 |
|  | (-4.13 - 3.84) | (-3.37 - 0.14) |
| Level change, phase 2 vs. 1 | 20.28** | 23.58** |
|  | (9.28 - 31.27) | (13.75 - 33.41) |
| Trend change, phase 2 vs. 1 | -1.38 | 0.36 |
|  | (-7.83 - 5.08) | (-4.34 - 5.05) |
| Level change, phase 3 vs. 2 | 5.86 | 5.83 |
|  | (-4.84 - 16.57) | (-3.20 - 14.87) |
| Trend change, Phase 3 vs. 2 | 2.61 | 2.61 |
|  | (-2.24 - 7.47) | (-1.79 - 7.02) |
| Phase 0 level | 15.41** | 2.36 |
|  | (9.50 - 21.31) | (-1.23 - 5.95) |
|  |  |  |
| Observations | 981 | 981 |
| Number of facilities | 34 | 34 |
| NHMIS = Nigerian Health Management Information System  Notes: All models are linear GEE models with robust standard errors adjusted for clustering at the facility level and using an exchangeable working correlation structure. Coefficients of models 3-5 are on percentages/ percentage point differences. Robust 95% confidence interval in parentheses. Trends are interpreted as the mean month-on-month change in the outcome at the facility-level. | | |

**Table S2. Sensitivity analyses: ITSA estimates combining Phases 0 and 1**

|  | (Model 1) | (Model 2) | (Model 3) |
| --- | --- | --- | --- |
|  | DMPA-SC SI | DMPA-SC PA | DMPA-SC (SI and PA) |
|  |  |  |  |
| Combined phases 0 and 1 trend | 0.14** | 0.18 | 0.32* |
|  | (0.05 - 0.22) | (-0.07 - 0.44) | (0.06 - 0.58) |
| Level change, Phase 2 vs. combined Phases 0 and 1 | 23.11** | -0.76 | 22.35** |
|  | (13.27 - 32.96) | (-5.63 - 4.10) | (11.43 - 33.27) |
| Trend change, Phase 2 vs. combined Phases 0 and 1 | 1.27 | -3.85** | -2.58 |
|  | (-2.00 - 4.53) | (-6.19 - -1.52) | (-6.44 - 1.27) |
| Level change, phase 3 vs. 2 | 4.05 | 0.93 | 4.98 |
|  | (-2.85 - 10.96) | (-4.64 - 6.49) | (-3.36 - 13.33) |
| Trend change, Phase 3 vs. 2 | -0.17 | 4.40** | 4.24* |
|  | (-3.56 - 3.22) | (2.05 - 6.76) | (0.17 - 8.30) |
| Phase 0 level | -0.35 | 15.64** | 15.29** |
|  | (-5.07 - 4.37) | (11.41 - 19.86) | (7.30 - 23.28) |
|  |  |  |  |
| Observations | 1,224 | 1,224 | 1,224 |
| Number of facilities | 36 | 36 | 36 |
| Notes: All models are linear GEE models with robust standard errors adjusted for clustering at the facility level and using an exchangeable working correlation structure. Adjusted models include variables for season (modeled as dummy variables indicating January-March, April-June, July-September, and October-December) and local governmental authority (LGA). Coefficients of models 3-5 are on percentages/ percentage point differences. Robust 95% confidence interval in parentheses. Trends are interpreted as the mean month-on-month change in the outcome at the facility-level. | | | |
| ** p<0.01, * p<0.05 |  |  |  |

**Table S3. Exploratory analyses: ITSA estimates by State**

| **Panel A. Mean Number of DMPA-SC Visits (PA and SI)** | | |  |
| --- | --- | --- | --- |
|  | (Model 1) | (Model 2) | (Model 3) |
|  | Lagos | Niger | Oyo |
|  |  |  |  |
| Phase 0 trend | 0.89** | -0.02 | 0.46* |
|  | (0.30 - 1.49) | (-0.42 - 0.39) | (0.02 - 0.91) |
| Level change, Phase 1 vs. 0 | -12.08** | 7.93 | -3.50 |
|  | (-16.63 - -7.53) | (-1.93 - 17.78) | (-11.32 - 4.32) |
| Trend change, Phase 1 vs. 0 | 0.15 | -0.44 | -4.59 |
|  | (-4.12 - 4.43) | (-4.27 - 3.38) | (-9.71 - 0.53) |
| Level change, phase 2 vs. 1 | 20.71** | 41.76** | 21.95* |
|  | (7.34 - 34.07) | (22.50 - 61.02) | (3.53 - 40.37) |
| Trend change, phase 2 vs. 1 | -3.75 | -5.21 | 5.71* |
|  | (-8.02 - 0.52) | (-16.35 - 5.94) | (0.15 - 11.26) |
| Level change, phase 3 vs. 2 | 4.60 | 5.57 | 5.03 |
|  | (-8.38 - 17.57) | (-8.88 - 20.03) | (-11.87 - 21.94) |
| Trend change, Phase 3 vs. 2 | 4.61 | 9.60 | -1.80 |
|  | (-0.52 - 9.74) | (-0.03 - 19.23) | (-5.91 - 2.32) |
| Phase 0 level | 4.28 | 19.03** | 19.32** |
|  | (-13.18 - 21.74) | (11.15 - 26.90) | (9.25 - 29.38) |
|  |  |  |  |
| Observations | 408 | 408 | 408 |
| Number of facilities | 12 | 12 | 12 |
| **Panel B. Mean Number of DMPA-SC SI Visits** | |  |  |
|  | (Model 1) | (Model 2) | (Model 3) |
|  | Lagos | Niger | Oyo |
|  |  |  |  |
| Phase 0 trend | 0.11* | 0.13 | 0.19 |
|  | (0.01 - 0.21) | (-0.08 - 0.34) | (-0.03 - 0.42) |
| Level change, Phase 1 vs. 0 | -2.41* | 3.85 | 2.10 |
|  | (-4.61 - -0.21) | (-0.22 - 7.92) | (-2.95 - 7.14) |
| Trend change, Phase 1 vs. 0 | 0.35* | -0.34 | -4.03* |
|  | (0.01 - 0.69) | (-2.16 - 1.48) | (-7.27 - -0.78) |
| Level change, phase 2 vs. 1 | 9.87** | 43.45** | 24.23** |
|  | (5.24 - 14.51) | (22.15 - 64.74) | (10.41 - 38.06) |
| Trend change, phase 2 vs. 1 | 2.17 | -1.46 | 7.08** |
|  | (-2.07 - 6.40) | (-9.71 - 6.80) | (2.99 - 11.17) |
| Level change, phase 3 vs. 2 | 6.58 | 1.31 | 4.24 |
|  | (-2.80 - 15.96) | (-11.21 - 13.83) | (-10.07 - 18.56) |
| Trend change, Phase 3 vs. 2 | -1.07 | 4.49 | -3.94 |
|  | (-5.13 - 2.99) | (-3.21 - 12.20) | (-8.57 - 0.69) |
| Phase 0 level | 3.38 | 1.05 | 2.19 |
|  | (-11.38 - 18.14) | (-7.08 - 9.17) | (-2.36 - 6.74) |
|  |  |  |  |
| Observations | 408 | 408 | 408 |
| Number of facilities | 12 | 12 | 12 |
| Notes: Models are linear GEE models with robust standard errors adjusted for clustering at the facility level and using an exchangeable working correlation structure and fit within subgroups defined by State. All models include variables for season (modeled as dummy variables indicating January-March, April-June, July-September, and October-December) and local governmental authority (LGA). Coefficients interpreted as the mean difference in visits at the facility/month-level. Robust 95% confidence interval in parentheses. Trends are interpreted as the mean month-on-month change in number of visits per month the facility-level. | | | |
| ** p<0.01, * p<0.05 |  |  |  |

**Figure S1: DMPA-SC SI training behavior change conceptual framework**


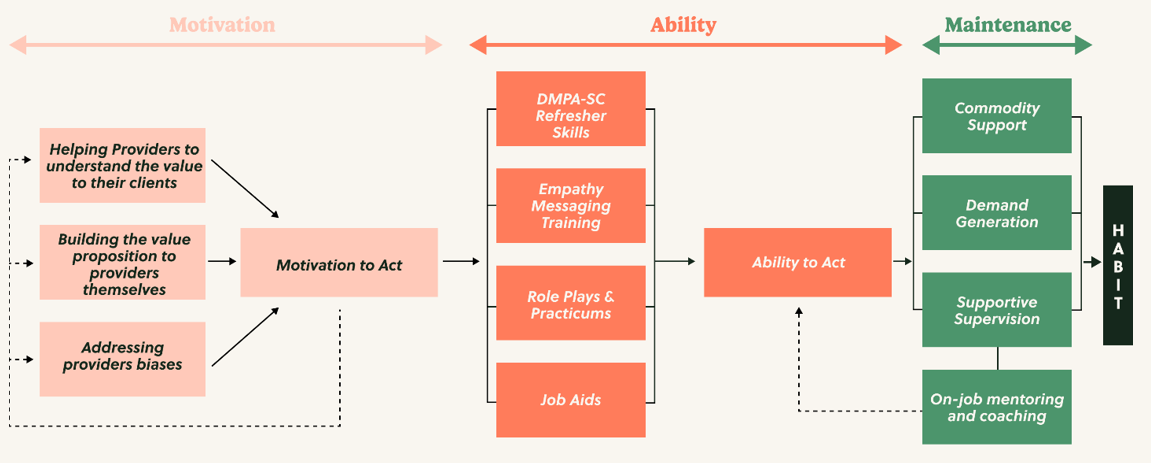


**Figure S2: Pre-, During-, and Post-Training Activities**


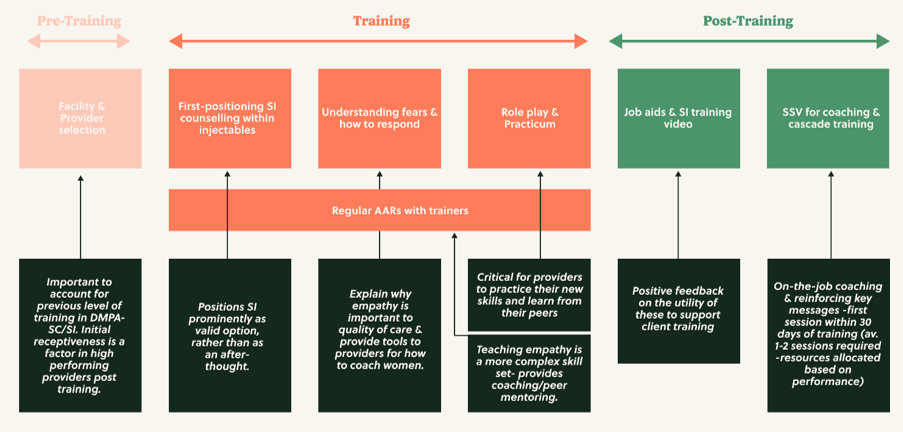


*AAR = after action review

**Figure S3. ITSA estimates for total FP visits**

Notes: Figures represent unadjusted ITSA models

**Figure S4. Sensitivity analysis: ITSA using HMIS data only**

**Panel A. Total DMPA-SC visits**

**Panel B. DMPA-SC SI visits**

Notes: Figures represent unadjusted ITSA models
